# Lost in Translation: Ribosome-Associated mRNA and Protein Quality Controls

**DOI:** 10.3389/fgene.2018.00431

**Published:** 2018-10-04

**Authors:** Andrey L. Karamyshev, Zemfira N. Karamysheva

**Affiliations:** ^1^Department of Cell Biology and Biochemistry, Texas Tech University Health Sciences Center, Lubbock, TX, United States; ^2^Department of Biological Sciences, Texas Tech University, Lubbock, TX, United States

**Keywords:** RNA quality control, protein quality control, post-transcriptional regulation of gene expression, RNA stability, RNA degradation, translation, protein targeting and folding, ribosome

## Abstract

Aberrant, misfolded, and mislocalized proteins are often toxic to cells and result in many human diseases. All proteins and their mRNA templates are subject to quality control. There are several distinct mechanisms that control the quality of mRNAs and proteins during translation at the ribosome. mRNA quality control systems, nonsense-mediated decay, non-stop decay, and no-go decay detect premature stop codons, the absence of a natural stop codon, and stalled ribosomes in translation, respectively, and degrade their mRNAs. Defective truncated polypeptide nascent chains generated from faulty mRNAs are degraded by ribosome-associated protein quality control pathways. Regulation of aberrant protein production, a novel pathway, senses aberrant proteins by monitoring the status of nascent chain interactions during translation and triggers degradation of their mRNA. Here, we review the current progress in understanding of the molecular mechanisms of mRNA and protein quality controls at the ribosome during translation.

## Introduction

**FIGURE 1:**
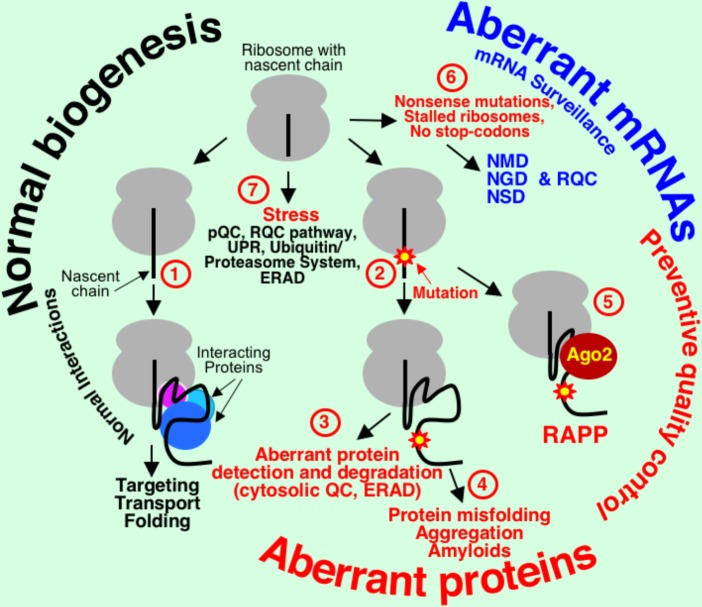
mRNA and protein quality control pathways in mammalian cells. Normal interactions of the nascent chains lead to proper protein transport/folding (1). Loss of these interactions due to defect in the interacting factor or mutation in the polypeptide nascent chain (2) leads to protein degradation (3), misfolding, aggregation, and amyloid formation (4), or mRNA elimination in the RAPP pathway (5). mRNA surveillance quality control systems (6), nonsense-mediated decay (NMD), non-stop decay (NSD), and no-go decay (NGD) detect and eliminate defective mRNAs with PTCs, mRNAs without natural stop codon, and mRNAs at the stalled in translation ribosomes, respectively. Nascent chains at the stalled ribosomes or during stress are ubiquitinated in the ribosome quality control complex (RQC) pathway and removed by proteasome. During ER stress pre-emptive quality control (pQC) cotranslationally reroutes secretory and membrane proteins to cytoplasm for degradation. Many proteins are misfolded during stress and they are removed by multiple cellular systems, like UPR, ERAD, and ubiquitin/proteasome system (7).

Genetic information is transferred during transcription and translation into correctly folded active proteins that are localized at the proper places for their functioning. Despite the high fidelity of these mechanisms, defective proteins can be produced as result of mutations, mistakes in transcription and translation, stress, or other reasons. Cellular quality control pathways evolved to prevent synthesis of the aberrant proteins at the ribosome or degrade them if they are already synthesized (**Figure 1**).Many quality control systems are engaged cotranslationally and conduct mRNA and protein surveillance at the ribosome. Protein synthesis and degradation of defective proteins are energetically expensive processes and ribosome-associated quality control can prevent futile aberrant protein synthesis. Nonsense-mediated decay (NMD), no-go decay (NGD), and non-stop decay (NSD) recognize and eliminate mRNAs with premature termination codons (PTCs), truncated and stalled in translation mRNAs, and mRNAs without natural stop codons, respectively (Welch and Jacobson, 1999; Doma and Parker, 2007; Shoemaker and Green, 2012; Popp and Maquat, 2013). Truncated polypeptides produced at the stalled ribosomes are ubiquitinated and degraded by proteasome (Dimitrova et al., 2009; Bengtson and Joazeiro, 2010; Brandman et al., 2012; Duttler et al., 2013; Brandman and Hegde, 2016). The regulation of aberrant protein production (RAPP) pathway senses aberrant proteins by scanning the status of nascent chains interactions during translation and triggers degradation of their mRNAs (Karamyshev et al., 2014; Pinarbasi et al., 2018). When defective mRNAs and proteins are missed by these quality control systems, the aberrant proteins are degraded by proteolytic machinery in the cytosol (Heck et al., 2010), or in the endoplasmic reticulum (ER) by ER associated degradation (ERAD) pathway (Brodsky and Wojcikiewicz, 2009). If aberrant proteins escape the quality control, they may misfold, form insoluble aggregates or amyloids, and result in many human diseases (Stefani and Dobson, 2003; Gregersen et al., 2006; Zimmermann et al., 2006; Hebert and Molinari, 2007; Jarjanazi et al., 2008; Hipp et al., 2014).

The interactions of a polypeptide nascent chain during translation have a crucial role in protein biogenesis and quality control (Gandin and Topisirovic, 2014). These interactions determine the future localization of the proteins, their folding and modifications (Pechmann et al., 2013). Disruptions of these processes may serve as signals for quality control machinery and for detection of abnormal mRNAs/proteins. In this review, we analyze nascent chain interactions occurring at the ribosome and the events taking place during ribosome-associated mRNA and protein quality controls.

## Nascent Chain Interactions During Translation are Important for Protein Targeting and Folding

Protein targeting, transport, and folding occur cotranslationally or posttranslationally (Park and Rapoport, 2012; Ellgaard et al., 2016). In this review, we focus only on co-translational protein interactions. During the first steps of translation, polypeptides exposed from the ribosomal exit tunnel start their first interactions with different factors required for folding, modification, targeting, and transport. Loss of these interactions leads to improper folding and protein degradation, protein aggregation and the formation of amyloids, or mRNA elimination (**Figure 1**). All living cells have different compartments and proteins should be precisely delivered to the proper locations in the cells. While cytosolic proteins remain in the cytosol after completing their synthesis, other proteins are transported to different cellular organelles or outside of the cell.

Despite very big differences between prokaryotic and eukaryotic cells, protein targeting and transport are regulated by similar mechanisms. Proteins possess specific localization signals that are recognized by specialized proteins (Emanuelsson and von Heijne, 2001). These interactions are essential for protein targeting. The best studied localization signals so far are signal sequences (von Heijne, 1985, 1990; Nilsson et al., 2015). Secretory proteins are synthesized as precursors containing N-terminal extension called signal sequence or signal peptide. Signal sequences are responsible for directing secretory proteins to Sec61 translocon in the ER membrane (in eukaryotes) or to SecYEG complex in the bacterial plasma membrane (in prokaryotes) for translocation through the membranes (Alder and Johnson, 2004; Wild et al., 2004; Egea et al., 2005; Rapoport, 2007; Dudek et al., 2015; Voorhees and Hegde, 2016). Different signal sequences do not have sequence homology, but possess similar structural features (von Heijne, 1985, 1990).

In bacteria, sorting events are determined by a balance of interactions of a newly synthesized nascent chain with Ffh/4.5S RNA complex, SecA protein, chaperone trigger factor, and other proteins (Karamyshev and Johnson, 2005; Eisner et al., 2006). Overproduction of secretory proteins leads to imbalance of targeting/folding and accumulation of their precursors in insoluble form in cytoplasm in bacteria (Nesmeyanova et al., 1991; Nesmeyanova et al., 1997).

In eukaryotic cells, the interactions of nascent chains are more complex and reflect more complicated process of cotranslational folding and targeting to multiple organelles. Signal sequences are recognized by signal recognition particle (SRP) (Walter et al., 1981; Krieg et al., 1986; Kurzchalia et al., 1986). These interactions serve as basis for cotranslational targeting of secretory proteins to translocon. In the case of membrane proteins, their first transmembrane spans are also recognized by SRP for targeting.

There are other localization signals for direction of the proteins to mitochondria, nucleus, and peroxisome (Emanuelsson and von Heijne, 2001). These signals are important for proper recognition by specialized targeting factors. Some of these signals are localized at the N-termini and thus probably are recognized cotranslationally, and some are at the C-terminus of the protein, suggesting posttranslational targeting. Examples of N-terminal signals include specialized mitochondrial presequences that enriched in positively charged residues and have ability to form amphiphilic α-helices (von Heijne et al., 1989; Emanuelsson and von Heijne, 2001), peroxisomal targeting signals type 2 (PTS2) for some peroxisomal proteins (Williams, 2014), and others. Tail-anchored (TA) proteins as well as PTS1 peroxisomal proteins contain C-terminal signals and most likely are targeted by posttranslational mechanisms (Stefanovic and Hegde, 2007; Williams, 2014).

**FIGURE 2:**
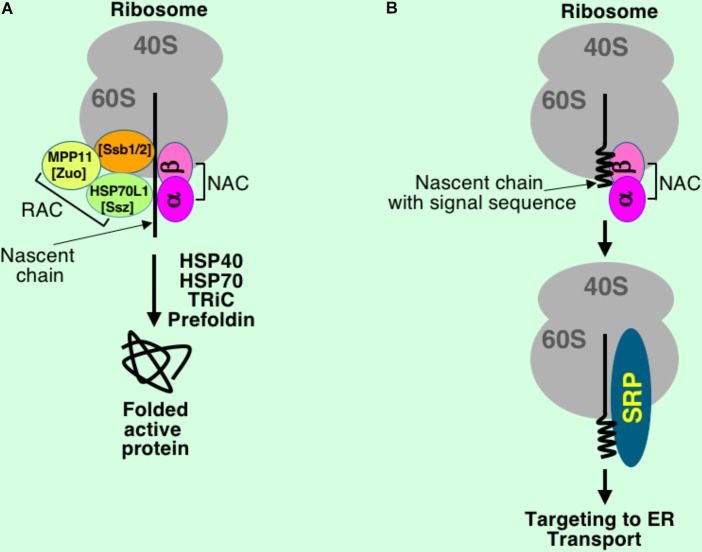
Simplified scheme of interactions at the polypeptide exit site at the ribosome under normal conditions. Nascent chains and ribosomes interact with different proteins during translation to achieve proper folding and correct targeting. While nascent chains of cytosolic proteins **(A)** are synthesized in environment of RAC, NAC, Ssb (HSP70) and further folded with assistance of chaperones and chaperonins, the secretory proteins **(B)** briefly interact with NAC before full exposure of the signal sequence, and when signal sequence is emerged from the ribosome tunnel, SRP binds it leading to temporary elongation arrest and targeting to the ER membrane for further transport through translocon into ER lumen, then to Golgi, and finally outside of the cell. Only major interacting partners are shown, their sizes are not to scale, and their positions on the ribosome and contacts are presented for a general understanding of the process and do not reflect very complex nature of their interactions with the nascent chains and the ribosomes. Mammalian proteins are shown, their yeast counterparts are in square brackets.

There are many specialized proteins that interact with nascent chains during their synthesis. They include targeting factors, chaperones assisting protein folding, and modification factors. These proteins are organized in a group with a general name ribosome-associated protein biogenesis factors (RPBs) (Raue et al., 2007). Nascent chains of cytosolic and secretory proteins interact with different partners of RPBs to achieve proper folding and correct targeting (**Figure 2**). RPBs act during translation when a short nascent chain emerges from the ribosomal polypeptide tunnel. In yeast, RPBs consist of targeting factor SRP, nascent polypeptide-associated complex (NAC), chaperones Ssb1 and Ssb2 (Hsp70 homologs), the ribosome-associated complex (RAC), N-terminal acetyltransferase (NatA), and Map1 and Map2 proteins (Raue et al., 2007).

Yeast RAC consists of two proteins, zuotin (or Zuo1, DnaJ homolog, Hsp40 family) and Ssz1p (DnaK homolog, Hsp70 family) (Gautschi et al., 2001; Zhang et al., 2017). Mammalian RAC includes chaperones MPP11 and HSP70L1 (Otto et al., 2005). It was found that RAC binds ribosomes near polypeptide tunnel exit (Peisker et al., 2008). NAC consists of two subunits, α and β, both of them are localized in close proximity to a nascent chain, as it was demonstrated by crosslinking (Wiedmann et al., 1994; Wang et al., 1995). It binds short nascent chains when they are just exposed from the polypeptide tunnel. In the case of secretory proteins, NAC binds the nascent chain only when signal sequence is not completely exposed from the ribosome (**Figure 2B**). Binding of NAC is important for SRP specificity and translocation fidelity (Wiedmann et al., 1994). In normal conditions, NAC binds ribosomes to promote protein folding, however in stress it moves to protein aggregates and functions as a protein chaperone (Kirstein-Miles et al., 2013). Chaperone Ssb binds wide variety of substrates – cytosolic, ER, nuclear, and mitochondria nascent polypeptides (Doring et al., 2017). Its binding accelerates translation. Ssb (Ssb1 and Ssb2), RAC, and NAC have a dual function in folding of new proteins and regulation of the ribosome production (Koplin et al., 2010).

It was also found that there are two major chaperone groups or networks with discrete functions in the cells, one is for *de novo* folding (named CLIPS for chaperones linked to protein synthesis) and the other (HSPs, heat shock proteins) is for protein refolding to rescue them in stress (Albanese et al., 2006). Thus, translation-associated chaperones are organized in the CLIPS network (Albanese et al., 2010; Pechmann et al., 2013). While secretory/membrane proteins need SRP during the first step of protein synthesis, cytosolic proteins require ribosome bound chaperones Ssb (HSP70 family) in yeast (Willmund et al., 2013), HSP70L1 and MPP11 in mammals (Otto et al., 2005), chaperonin TRiC (McCallum et al., 2000; Etchells et al., 2005), and other factors (Hartl et al., 2011; Pechmann et al., 2013). Another chaperonin, prefoldin, also binds nascent chains and is involved in folding of actin and tubulin (Hartl and Hayer-Hartl, 2002). It is not completely understood how specificity of chaperones/chaperonins to nascent chains is controlled. In addition, large group of proteins involved in quality control and ubiquitination of aberrant nascent chains are also found bound to translating ribosomes (Comyn et al., 2014).

Thus, ribosome itself serves not only as a protein synthesis machinery but it also plays a key role in arranging protein targeting/folding and quality control. Studying the normal interactions of nascent polypeptides during translation and their change during engagement of mRNA and protein quality control machineries are important for understanding of molecular foundation of protein biogenesis and homeostasis, as well as for molecular basis of human diseases associated with dysregulation of these processes.

## Ribosome-Associated mRNA Quality Control Pathways

**FIGURE 3:**
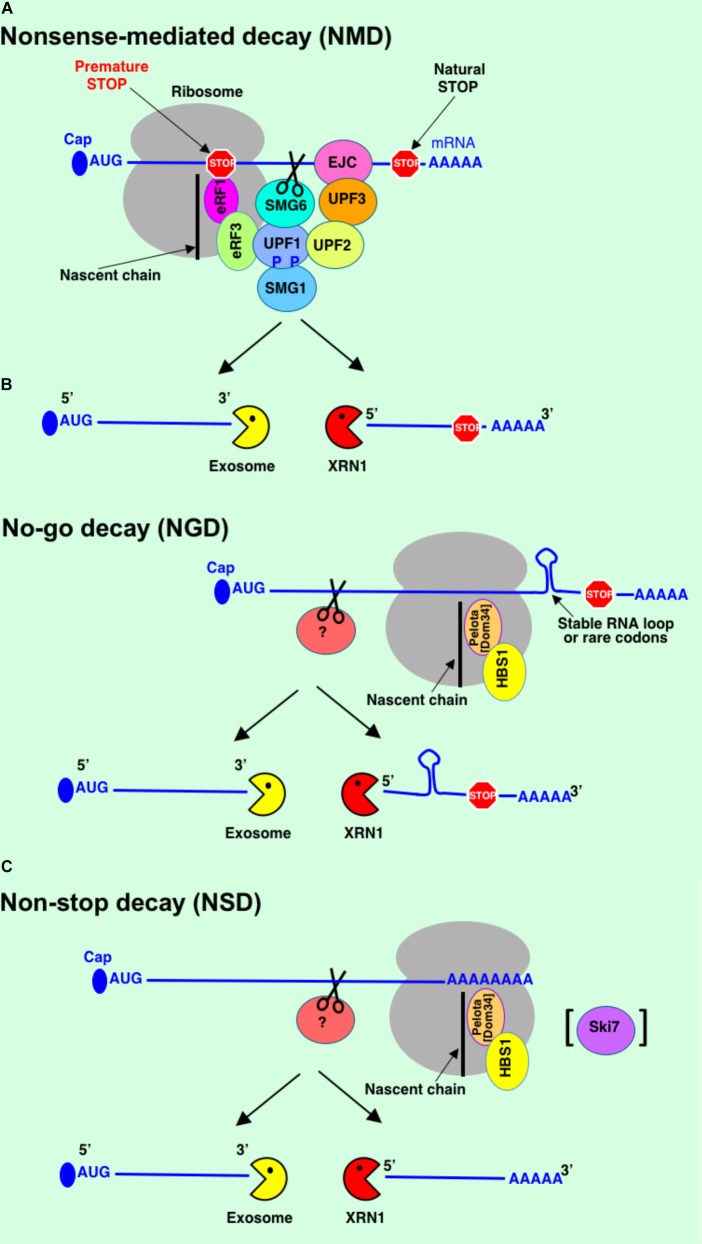
mRNA quality control systems: Nonsense-mediated decay (NMD) **(A)**, no-go decay (NGD) **(B)**, non-stop decay (NSD) **(C)**. See text for details.

mRNA turnover is one of the major mechanisms to control gene expression and maintain a high level of fidelity for cell function and viability. Cells use multiple mRNA degradation pathways to eliminate non-functional transcripts. mRNA decay is a highly orchestrated process controlled by distinct set of genes. mRNA surveillance starts in the nucleus. Defective mRNAs could be detected and subjected for degradation at different stages of their production and maturation including transcription, capping, splicing, and polyadenylation. Exosome is the major machinery to degrade the faulty mRNAs in the nucleus. Then mRNAs that passed a quality control in the nucleus are exported to the cytoplasm as messenger ribonucleoproteins (mRNPs) where they can be engaged in translation. In the cytoplasm, mRNAs are subjected to additional cotranslational mRNA surveillance quality control. Several major mRNA degradation pathways operate to identify faulty mRNAs and protect the cell from translation of aberrant mRNAs and potentially toxic proteins – NMD, NGD, and NSD (**Figure 3**).

## Nonsense-Mediated Decay

Nonsense-mediated decay is mRNA surveillance pathway that recognizes and targets mRNAs with PTCs for rapid degradation to reduce translation of truncated proteins with dominant-negative or deleterious gain-of-function activities (Welch and Jacobson, 1999; Popp and Maquat, 2013) (**Figure 3A**). This pathway exists in all eukaryotes examined so far (Culbertson, 1999). NMD was not found in bacteria. The presence of the PTCs in bacterial genes leads to termination or reinitiation of translation (Karamyshev et al., 2004).

Exon-exon junction complex (EJC) is a complex of proteins that are assembled at the pre-mRNA during splicing (Gehring et al., 2009). After mRNA export EJC is being removed from the mRNA during pioneer round of translation and replaced with proteins promoting translation. However, if premature termination codon is present on the mRNA ≥ 50–55 nucleotides upstream of the EJC the NMD is activated most likely because the terminating ribosome (at the PTC) is not able to remove EJC and proceed with normal translation (Popp and Maquat, 2014).

Several proteins are conserved in NMD across species and constitute the core of this pathway: the up-frameshift proteins UPF1, UPF2, and UPF3. UPF1 is the master regulator of NMD. ATPase activity of UPF1 is required for disassembly of mRNPs during NMD (Franks et al., 2010). In mammals, two variants of UPF3 exist: UPF3a and UPF3X (UPF3b) (Serin et al., 2001). In multicellular organisms, additional proteins called suppressors with morphological effects on genitalia (SMG1, SMG5 – SMG9) contribute to the regulation of NMD (Yamashita et al., 2001, 2009). NMD takes place in three stages including detection of NMD substrates, tagging, and finally degradation of PTC containing transcript. NMD activation begins with detection of PTC during pioneer round of translation. After detection stage the PTC is tagged by formation of SURF complex at the terminating ribosome. SURF complex includes the serine/threonine kinase SMG1, UPF1, and eukaryotic release factors eRF1-eRF3 (Kashima et al., 2006; Hwang et al., 2010). Then UPF1-SMG1 binds to EJC via interaction with UPF2. UPF2 is bound to EJC through interaction with UPF3 or UPF3X. SMG1 phosphorylates UPF1. Hyperphosphorylated UPF1 induces translational repression and recruits SMG6 protein (Isken et al., 2008). SMG6 performs endonucleolitic cleavage of mRNA. This cleavage occurs between the PTC and EJC sites of the defective mRNA during last stage of NMD (Huntzinger et al., 2008; Eberle et al., 2009). Activated UPF1 then recruits SMG5-SMG7 or SMG5-PNRC2 (Kervestin and Jacobson, 2012). These proteins further recruit decapping and/or deadenylation machinery to facilitate exonucleolytic degradation of unprotected 5′- and 3′-mRNA fragments resulted from endonucleolytic cleavage of PTC-containing mRNA (Lejeune et al., 2003; Loh et al., 2013). 5′-to-3′ exonuclease XRN1 is responsible for degradation of the 3′-cleavage product (Lejeune et al., 2003; Unterholzner and Izaurralde, 2004; Eberle et al., 2009). The 5′-cleavage product most likely is degraded by exosome (Schmid and Jensen, 2008). NMD proteins can be co-purified with components of mRNA degradation machinery (DCP2, XRN1, and XRN2/RAT1, and several exosome subunits) (Lejeune et al., 2003; Muhlemann and Lykke-Andersen, 2010). Decapping and deadenylation enzymes may contribute to faster degradation of mRNA fragments in mammalian cells (Lejeune et al., 2003). However, more research is needed to understand the role of decapping and deadenylation in NMD.

While the mechanism explained above (Exon Junction Complex model) is appealing, it cannot explain all the details of the NMD mechanism and alternative models including Upf1 3′-UTR sensing/potentiation and the faux 3′-UTR models were proposed (reviewed in He and Jacobson, 2015). While the models recognize importance of 3′-UTR, however, they propose different roles for 3′-UTR and NMD target recognition (Amrani et al., 2004; Hogg and Goff, 2010). According to sensing/potentiation model Upf1 senses 3′-UTR and potentiates mRNA decay (Hogg and Goff, 2010). According to faux model, efficient termination is inhibited when the distance between PTC and polyA tail is large (Amrani et al., 2004).

The major function of UPF1, the master regulator of NMD, is to limit translation from aberrant mRNAs. Thus, NMD is translation-dependent process and truncated protein derived from pioneer round of translation could be toxic and contribute to human pathology. Therefore, PTC-containing mRNA degradation should be coupled to the protein degradation of truncated polypeptide. While limited information is available in this regard some studies on yeast suggest that Upf1 could have E3 ubiquitin ligase properties promoting degradation of truncated polypeptide through proteasome (Takahashi et al., 2008; Kuroha et al., 2009). However, the fate of truncated proteins produced during NMD in mammalian cells remains an open question for further investigations.

## No-Go Decay

No-go decay degrades mRNAs stalled in translation elongation complexes (**Figure 3B**). Translational arrest could be caused either by specific features of nascent peptides, strong secondary structures in mRNA physically blocking the translation machinery along the transcript, or a rare codon repeat causing the A site to be unoccupied for a long duration (Kuroha et al., 2010; Tsuboi et al., 2012). Insertion of stable stem-loop RNA structure into *PGK1* mRNA led to translational arrest and endonucleolytic cleavage of mRNA stalled in translation elongation with subsequent rapid mRNA degradation. While NGD pathway was initially discovered in yeast, it was also identified in fruit flies and mammals (Doma and Parker, 2006; Passos et al., 2009; Pisareva et al., 2011).

Proteins Pelota (in mammals; Dom34 in yeast) and HBS1 are involved in regulation of NGD pathway (Doma and Parker, 2006; Pisareva et al., 2011) and are structurally related to the termination factors eRF1 and eRF3, respectively (Atkinson et al., 2008). They also mimic complex of elongation factor and tRNA suggesting that they bind A site at the ribosome (van den Elzen et al., 2010). Indeed, Dom34 and Hbs1 interact directly with A site of the ribosome but instead of termination they promote dissociation of aberrant translation elongation complex and ribosome recycling (Shoemaker et al., 2010; Becker et al., 2011). Dom34/Hbs1 can also stimulate endonucleolytic cleavage event in NGD substrate and promote subsequent mRNA degradation, however, these factors are not essential since cleavage of NGD mRNA can take place even in the absence of these proteins (Passos et al., 2009; Tsuboi et al., 2012). The data suggest that endonucleolytic cleavage occurs upstream of the ribosome stalling site (Tsuboi et al., 2012). It was shown recently that NGD is triggered by the ribosome collision resulting in multiple endonuclease cleavages (Simms et al., 2017). Efficiency of NGD depends on the ribosome density on the substrate mRNA suggesting that ribosome collision transmits signal to activate endonuclease. Like in NMD pathway, generated fragments are rapidly degraded by the exosome and XRN1 during NGD. It still remains unknown what endonuclease is responsible for the cleavage of NGD substrates.

## Non-Stop Decay

Non-stop decay degrades mRNAs that lack stop codons (**Figure 3C**). NSD was first discovered in yeast (van Hoof et al., 2002) and mammals (Frischmeyer et al., 2002). Non-stop mRNAs could arise by different mechanisms. These aberrant mRNAs may be produced by erroneous polyadenylation within the ORF resulting in non-stop mRNAs with poly(A) or by endonucleolytic cleavage within the ORF generating non-stop mRNA lacking poly(A) (Ozsolak et al., 2010; Graille and Seraphin, 2012). Translation of poly(A) leads to formation of poly-lysine chain at the C-terminus of the synthesized polypeptide. This positively charged amino acid chain causes stalling of the polypeptide in the ribosome tunnel most likely due to interaction with negatively charged ribosomal RNA (Dimitrova et al., 2009). In case of truncated non-stop mRNAs lacking poly(A), ribosomes stall at the very 3′-end of the mRNA. In both cases, translational stalling triggers rapid degradation of non-stop mRNAs by the translation-dependent NSD pathway. Translational repression is a prerequisite for mRNA degradation during NSD (Inada and Aiba, 2005; Akimitsu et al., 2007) similarly to NGD and NMD. It was shown that Ski7, a protein structurally related to Hbs1 and eRF3, is able to bind stalled ribosome and recruit exosome to the transcript during NSD in yeast (van Hoof et al., 2002). However, Ski7 is not present in higher eukaryotes. Organisms lacking Ski7 rely on Hbs1 and Dom34 proteins that function in both NSD and NGD (Tsuboi et al., 2012; Saito et al., 2013) suggesting a substantial overlap in function of these pathways. Recent study from Inada’s group has shown that Dom34:Hbs1 complex has a crucial role to dissociate ribosomes and stimulate mRNA degradation in both NSD and NGD pathways (Tsuboi et al., 2012). Endonucleolytic cleavage is a first step for mRNA degradation in NSD. It has been found that stalled ribosomes can induce multiple endonucleolytic cleavage events on non-stop mRNA covered by the individual ribosomes (Tsuboi et al., 2012). However, similar to NGD, the identity of endonuclease implicated in NSD is not known yet.

Thus, all of these cotranslational quality control systems share several common features: the aberrant mRNA must be eliminated, the truncated protein products should be degraded and the stalled ribosomes should recover from stalling and return for translation. NMD was originally discovered as a surveillance pathway with major function to reduce errors in gene expression by eliminating PTC-containing mRNAs; however, new roles of the NMD pathway have recently emerged. It has been found that NMD pathway is also capable to target normal and physiologically functional mRNAs in order to drive a rapid change in gene expression (He and Jacobson, 2015). Ribosome profiling revealed that the NMD pathway regulates expression levels of at least 10% of human transcripts (Celik et al., 2017). NMD contributes to regulation of germ granules and spermatogenesis, and NMD components were found in the composition of chromatoid body (Meikar et al., 2014; Bao et al., 2016; MacDonald and Grozdanov, 2017). It is conceivable that NSD and NGD pathways are also involved in regulation of gene expression in addition to mRNA quality control in a similar manner as NMD. Recent data suggest that NGD pathway can be used not only to degrade faulty mRNAs but also normal histone mRNAs from stalled degradation complexes as a part of cell cycle regulation (Slevin et al., 2014). Chemically damaged mRNAs (oxidized, depurinated, or alkylated) can cause translational stalls and become NGD substrates in order to reduce burden of toxic protein products for the cell (Shan et al., 2007; Wurtmann and Wolin, 2009).

Deficiencies in the NMD components such as UPF3B and SMG9 lead to an intellectual disability or multiple congenital anomaly syndrome, respectively, due to global transcriptional deregulation (Rebbapragada and Lykke-Andersen, 2009; Shaheen et al., 2016). The NMD pathway has also been found to regulate immune responses. The component of NMD, UPF1, is involved in antiviral responses and restricts the Semliki Forest virus (SFV) and Sindbis viral infections (Balistreri et al., 2014). Somatic mutations in *UPF1* gene are connected to pancreatic adenosquamous carcinoma (Liu et al., 2014). Deregulation of NMD pathway is associated with several types of cancer and reviewed in details in the recent publication (Popp and Maquat, 2018).

## Regulation of Aberrant Protein Production

**FIGURE 4:**
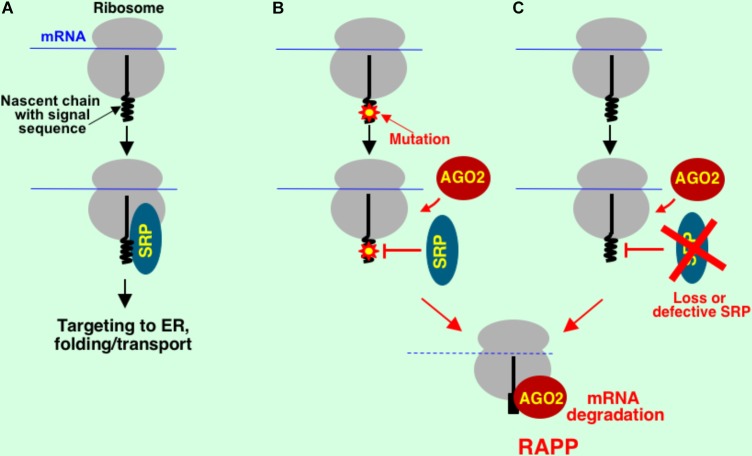
Model for regulation of aberrant protein production (RAPP) in mammalian cells. Normal cotranslational interactions are important for protein biogenesis. Nascent chains of secretory proteins interact with signal recognition particle (SRP). This interaction leads to proper protein targeting to ER, folding and transport **(A)**. Loss of this normal interaction with SRP due to a critical mutation in the secretory protein **(B)** or loss of the interacting factor **(C)** leads to engagement of AGO2 (a protein involved in translational silencing). This interaction directs aberrant protein mRNA for degradation initiating the RAPP process.

Novel type of ribosome-associated protein quality control, RAPP, was recently discovered (Karamyshev et al., 2014) (**Figures 1, 4**). The first natural RAPP substrate, granulin with disease-causing signal sequence mutations, was also recently identified, demonstrating that RAPP activation serves as a molecular mechanism for some types of frontotemporal lobar degeneration (Pinarbasi et al., 2018). The RAPP pathway detects aberrant proteins during translation and degrades their mRNA templates to prevent synthesis of potentially hazardous products (preventive quality control). It involves recognition of nascent chains that lost their normal interactions with factors for targeting and directs the aberrant protein mRNA for degradation. Original research was conducted on the example of secretory protein preprolactin with deletions in the signal sequence (Karamyshev et al., 2014). The central event of the RAPP pathway is a balance of interactions at the ribosome during translation. Normally, during translation, secretory proteins are recognized by SRP and targeted to the ER membrane for translocation through a translocon into the ER lumen (**Figure 4A**). When an aberrant signal sequence is not recognized by SRP due to a mutation or when SRP is absent or defective, AGO2 protein binds ribosome-nascent chain complex and triggers specific mRNA degradation (**Figures 4B,C**). Thus, SRP has a novel function in mRNA protection of the secretory proteins from degradation in addition to its role in protein targeting.

Although there are no distinct sequence requirements to trigger mRNA degradation, a mutation should take place in the vicinity of the region responsible for a necessary protein interaction and lead to impairment of this interaction. The AGO2 role in this process is not known yet. We hypothesize that the positioning of AGO2 close to a mutated nascent chain regulates its ability to direct mRNA for degradation. AGO2 is a protein that is involved in miRNA and siRNA response, translational silencing and a major component of RNA-induced silencing complex (RISC) (Hammond et al., 2001; Martinez et al., 2002). However, our experiments demonstrated that RAPP process does not involve miRNAs, Drosha and Dicer proteins suggesting a novel AGO2 function in the absence of RISC formation (Karamyshev et al., 2014). AGO2 possesses slicer or ribonuclease H activity (Liu et al., 2004; Song et al., 2004; Rivas et al., 2005). However, experiments involving enzymatically inactive AGO2 indicate that AGO2 slicer activity is not required for mRNA degradation during RAPP (Karamyshev et al., 2014). We have found that the mRNA degradation of the model RAPP substrates was suppressed by AGO2 depletion and accelerated by AGO2 overexpression. However, granulins with disease-causing mutations were not affected (Pinarbasi et al., 2018). These observations suggest that AGO2 functions as a sensor for some substrates during RAPP response, and an unidentified protein may serve that function for other substrates. Other explanation is that the major sensor of the pathway is not determined yet and AGO2 conducts a helper or enhancer function for some substrates. Our data suggest that the mRNA cleavage is conducted by other than AGO2 endonuclease. However, the nature of the endonuclease still remains to be found. Thus, the mechanism of the RAPP pathway is far from understanding yet.

It was found earlier that under stress conditions that lead to accumulation of unfolded proteins in ER, a process known as regulated Ire1-dependent decay (RIDD) is triggered (Hollien and Weissman, 2006; Hollien et al., 2009). It reduces quantity of secretory protein mRNAs to decrease accumulation of secretory proteins in ER during unfolded protein response (UPR). RIDD is an important general stress response mechanism that senses unfolded secretory proteins that have been successfully transported into ER, and prevents their further synthesis and therefore transport into ER and accumulation. By contrast, RAPP senses mutated polypeptide nascent chains that are not able to interact with SRP and therefore are not targeted and not translocated into ER thereby reducing accumulation of these potentially hazardous proteins in the cytosol.

The current RAPP model is based on selection of mRNA for degradation by a loss of cotranslational interaction between nascent chain and targeting factor at the ribosome. If interaction with SRP is reduced due to a mutation in the signal sequence then AGO2 interacts with nascent chain and directs its mRNA for degradation. If SRP interaction is intact, AGO2 cannot interact with nascent chain. It is possible that this mechanism is general and involved in quality control of other types of proteins that lost their normal interactions. It could be cytosolic aberrant proteins that lost natural interactions with some chaperones (for instance, ribosome associated chaperones and components of RAC, MPP11, and HSP70L1), or peroxisomal and mitochondrial proteins, that lost their interactions with their targeting factors. However, the understanding of these processes requires future studies.

## Ribosome-Associated Quality Control at a Nascent Chain Level

**FIGURE 5:**
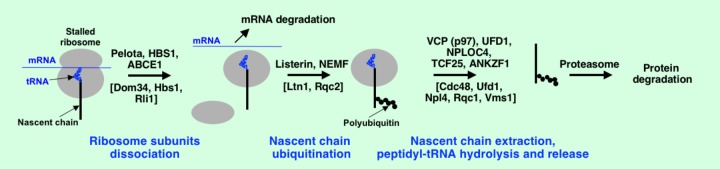
Protein quality control at the stalled ribosomes. Mammalian proteins are shown, their yeast counterparts are in square brackets. See text for details.

What happens to partially synthesized nascent chains after activation of degradation of the faulty mRNAs? Recent studies on cotranslational quality control systems induced by translational stalls have revealed that not only faulty mRNAs but also truncated proteins are rapidly degraded. In yeast, Ltn1, a ribosome-associated E3 ubiquitin ligase (Bengtson and Joazeiro, 2010) and a component of Ccr4-Not complex, Not4p (that may act as E3 ubiquitin-protein ligase) (Panasenko et al., 2006; Dimitrova et al., 2009; Halter et al., 2014) play important role in aiding of truncated protein products for degradation by proteasome. It was demonstrated that Ltn1, Tae2 (other name Rqc2), Rqc1, and AAA-ATPase Cdc48 (other names VCP, valosin containing protein, and p97) are involved in removal of aberrant translational products in yeast and form a complex on the 60S ribosome subunit (Brandman et al., 2012; Defenouillere et al., 2013). This complex was named the ribosome quality control complex (RQC) (Brandman et al., 2012). Listerin, the functional mammalian homolog of Ltn1, is involved in ubiquitination of aberrant nascent chains produced by the stalled ribosomes (Shao et al., 2013) (**Figure 5**). Notably, that the ubiquitinated nascent chains were found still attached to tRNAs, however, the process required dissociation of the ribosome subunits. Pelota, HBS1, ABCE1 are involved in the ribosome subunits dissociation in mammals, while Dom34, Hbs1, Rli1 are in yeast (Shoemaker et al., 2010; Pisareva et al., 2011; Shoemaker and Green, 2011) (**Figure 5**). Ribosome subunits dissociation leads to assembly of the RQC on the 60S ribosome subunit (Shao et al., 2015). Binding of nuclear export mediator factor (NEMF) in mammals (Rqc2 or Tae2 in yeast) prevents subunits association, leads to recruitment of Listerin and its positioning near the polypeptide exit site on the 60S subunit (Lyumkis et al., 2014; Shao et al., 2015). The results of several studies suggested that Cdc48, Npl4, Ufd1, and Rqc1 are involved in extraction of the ubiquitinated nascent chains from the 60S subunit (Brandman et al., 2012; Defenouillere et al., 2013; Verma et al., 2013). The mammalian orthologs are VCP (p97), UFD1, NPLOC4, and TCF25 (Verma et al., 2018). However, the detailed role of the distinct components is not well understood. It was discovered recently that yeast Vms1 (ANKZF1 in mammals) releases ubiquitinated nascent chains from the stalled ribosomes by peptidyl-tRNA hydrolysis for further degradation of polypeptides by proteasome (Verma et al., 2018). Very little is known about truncated nascent chain degradation during NMD pathway. It was shown that UPF1 promotes degradation of truncated peptides generated in NMD pathway and can potentially serve as E3 ubiquitin ligase (Takahashi et al., 2008; Kuroha et al., 2009). However, more research is needed to identify all key players in regulation of cotranslational protein degradation and details of the mechanism during NMD.

Several cotranslational protein quality controls induced by stress were recently discovered in mammals. One of them, pre-emptive quality control (pQC), cotranslationally reroutes membrane and secretory proteins to cytoplasm for degradation under acute ER stress (Kang et al., 2006; Kadowaki et al., 2015). Derlins (degradation in ER proteins) redirect them from the translocon to the proteasome with involvement of chaperone Bag6 (BCL2 associated athanogene 6) and p97 (alias Cdc48 or VCP) (Kadowaki et al., 2015). pQC reduces the burden of misfolded proteins in the ER during stress. Bag6 complex is also involved in mislocalized protein degradation pathway (Hessa et al., 2011). This pathway senses the presence of unprocessed or non-inserted hydrophobic domains released into the cytosol and directs these proteins for degradation. Other stress-induced quality control involves recruitment of c-Jun N-terminal kinase (JNK) to ribosomes by the receptor for activated protein C kinase 1 (RACK1), phosphorylation of elongation factor eEF1A2, and promotion of degradation by proteasome (Gandin et al., 2013). It implicates the complex JNK/RACK1/eEF1A2 in protein quality control at the ribosome in response to stress.

## Conclusion

Thus, nascent chains interact with a number of different factors at the ribosome during translation. These interactions are required for normal folding, transport and formation of active proteins. The alterations of these important interactions because of mutations or defective factors trigger protective mechanisms to prevent accumulation of the potentially toxic products in the cells. In addition, different aberrations in mRNAs may lead to translational stalling that prevents new rounds of translation and potentially may be fatal. Cells developed protective mechanisms to recycle stalled ribosome and remove aberrant proteins and mRNAs. Therefore, network of ribosome-associated proteins, endo- and exo-nucleases, chaperones, ubiquitin ligases, proteasome and other proteins, working in concert, is maintaining protein homeostasis in the cells. Multiple mechanisms are engaged at different stages of protein biogenesis to get rid of aberrant mRNA templates, mutated or uncompleted nascent chains, and misfolded or mislocalized proteins. However,many details of these mechanisms are still not completely understood and additional studies are needed to fill that gaps.

## Author Contributions

AK and ZK wrote, discussed, and edited the manuscript.

## Conflict of Interest Statement

The authors declare that the research was conducted in the absence of any commercial or financial relationships that could be construed as a potential conflict of interest.
